# Durable Disease Control with MEK Inhibition in a Patient with NRAS-mutated Atypical Chronic Myeloid Leukemia

**DOI:** 10.7759/cureus.414

**Published:** 2015-12-17

**Authors:** Vishesh Khanna, Scott T Pierce, Kim-Hien T Dao, Cristina E Tognon, David E Hunt, Brian Junio, Jeffrey W Tyner, Brian J Druker

**Affiliations:** 1 Knight Cancer Institute, Oregon Health & Science University; 2 Howard Hughes Medical Institute Medical Research Fellows Program, Howard Hughes Medical Institute; 3 Cleveland Clinic Lerner College of Medicine, Case Western Reserve University; 4 Department of Hematology/Oncology, Saint Joseph Hospital; 5 Saint Joseph Hospital Laboratory, Saint Joseph Hospital; 6 Howard Hughes Medical Institute Investigator Program, Howard Hughes Medical Institute; 7 Division of Hematology & Medical Oncology, Oregon Health & Science University; 8 Department of Cell, Developmental & Cancer Biology, Oregon Health & Science University

**Keywords:** atypical chronic myeloid leukemia, chronic neutrophilic leukemia, trametinib, nras

## Abstract

Atypical chronic myeloid leukemia (aCML) and chronic neutrophilic leukemia (CNL) are rare hematologic neoplasms characterized by leukocytosis and a hypercellular bone marrow. Although recurrent mutations in the colony-stimulating factor 3 receptor (*CSF3R*) are frequently observed in patients with (CNL), the mutational landscape in (aCML) is less well-defined. In this report, we describe an 81-year-old male who was diagnosed with aCML. He presented with leukocytosis and anemia but no significant clinical symptoms. Standard laboratory studies revealed the absence of the Philadelphia chromosome. Massively parallel sequencing demonstrated no mutations in *CSF3R*, but the presence of a heterozygous NRAS-G12D variant (47% allele frequency). The patient was started on treatment with trametinib, an MEK1/2 inhibitor with Food and Drug Administration approval for malignant melanoma. Therapy with trametinib resulted in exceptional improvements in his blood counts and continued disease control with 14 months of follow-up. This case highlights the need for clinical trials evaluating the safety and efficacy of MEK1/2 as a therapeutic target for the treatment of patients with *NRAS*-mutated aCML/CNL.

## Introduction

Atypical chronic myeloid leukemia (aCML) and chronic neutrophilic leukemia (CNL) are rare myeloid malignancies that exhibit overlapping clinical characteristics, including leukocytosis, anemia, thrombocytopenia, splenomegaly, and constitutional symptoms. These leukemias lack established standards of care and are associated with a poor prognosis [1]. Recent work has implicated mutations in colony-stimulating factor 3 receptor (*CSF3R*) as pathogenetic events in patients with CNL [2], but the molecular pathogenesis of aCML is more heterogeneous [3]. Of note, mutations in *NRAS* are reported to occur in up to one-third of patients with aCML [3]. One *NRAS* mutation, in particular, c.35G>A, results in the substitution of a glycine (G) to an aspartic acid (D) at position 12 in the NRAS protein, resulting in constitutive RAS activation due to disruption of RAS-GAP-mediated GTP hydrolysis [4]. This leads to downstream activation of the RAF-MEK-ERK pathway.

Trametinib (Novartis) is a reversible, allosteric inhibitor of MEK1/2, inhibiting both its intrinsic kinase activity and its phosphorylation by RAF [5]. It is currently FDA-approved for melanoma. Preclinical work has demonstrated the efficacy of trametinib in models of RAS-driven leukemias, *in vitro *and *in vivo*. For example, Jing, et al. showed that acute myeloid leukemia (AML) cell lines that harbor *NRAS*mutations are sensitive to trametinib inhibition [6]. Transplantation of Nras-G12D-positive AML cells into mice induces a lethal leukemia and treatment of these mice with trametinib significantly prolongs survival as compared to untreated control mice [7]. These studies indicate that targeting MEK with trametinib may represent a promising therapeutic strategy for the subset of patients with aCML whose leukemia harbors RAS mutations. 

In this report, we describe a patient with NRAS-G12D-positive aCML who experienced an exceptional response to MEK1/2 inhibition with trametinib.

## Case presentation

The Oregon Health and Science University Institutional Review Board approved this study; no protocol number was assigned as the IRB determined that it was exempt from requiring an associated number. Informed patient consent was obtained for treatment as well as for publication of the case.

An 81-year-old male with a history of coronary artery disease presented to the emergency department of a local hospital following a syncopal episode in April 2014. He was found to have a white blood cell (WBC) count of 54 x 10^3^ cells/µL. A prior CBC in July 2013 was within normal limits.

By May 2014, his WBC count had risen to 86 x 10^3^/µL, with an absolute neutrophil count (ANC) of 59.5 x 10^3^/µL. His hemoglobin (Hgb) was 9.8 g/dL, and his platelet count was 239 x 10^3^/µL. Peripheral blood smear demonstrated an overabundance of granulocytes with increased immature myeloid cells (> 10%), including 1% blasts. His bone marrow was 95% cellular with marked myeloid hyperplasia (myeloid:erythroid ratio = 10:1); there was a full maturation of the myeloid lineage with 1% blasts as well as the presence of megakaryocytic atypia. Flow cytometric analysis of his bone marrow revealed 89% neutrophilic cells, 6% monocytes, 2% lymphocytes, 1% eosinophils, 0.6% basophils, and 1% blasts.

Initially, the patient had mild symptoms consisting of a persistent nonproductive cough, slight fatigue, and minor lower extremity swelling. He denied other constitutional symptoms, such as weight loss, fevers, easy bruising, or early satiety. Physical examination was generally unremarkable with no appreciable splenomegaly. Standard diagnostic evaluation showed no evidence of BCR-ABL rearrangements or *JAK2* mutations. A blood sample was submitted for massively parallel sequencing to the Clinical Laboratory Improvement Amendments/College of American Pathologists-certified Knight Diagnostic Laboratories and showed no mutations in *CSF3R*, *MPL*, or *CALR*. It did, however, reveal the presence of an *NRAS* (c.35G>A; p.G12D) mutation at a 47% allele frequency and mutations in *SRSF2 *(p.P95R; 43% allele frequency) and *TET2* (p.K326fs*21; 48% allele frequency).

Three months after diagnosis, the patient began experiencing progressive fatigue, increasing lower extremity edema, and loss of appetite. His laboratory studies demonstrated a steady increase in the WBC count (256 x 10^3^/µl), with a Hgb of 9.9 g/dL, and a platelet count of 66 x 10^3^/µl. In addition, liver enzymes and serum creatinine were mildly elevated. Physical examination remained unremarkable, except for the observation of lower extremity edema, but no splenomegaly.

**Figure 1:**
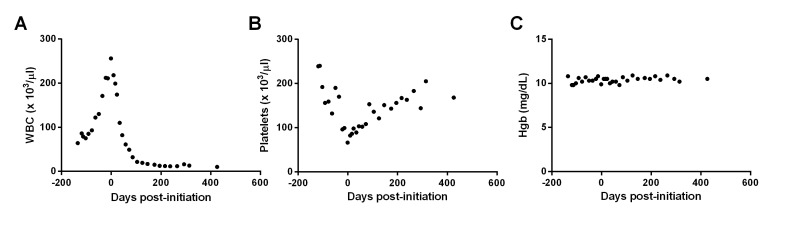
Response of hematologic parameters to trametinib treatment in a patient with NRAS-G12D-driven atypical CML. WBC (A), platelet (B), and Hgb (C) laboratory values prior to and after initiation of trametinib. Day 0 represents the first day of treatment.

The patient declined cytotoxic chemotherapy and, given the presence of the known *NRAS* mutation, was offered off-label treatment with trametinib. Treatment was initiated on September 10, 2014 (day 0), at 2 mg per day. As shown in Figure 1A, trametinib administration reduced the patient’s WBC counts rapidly. This stabilization of WBCs was accompanied by a reduction in immature granulocytes in his peripheral blood and normalization of ALT, AST, alkaline phosphatase, and serum creatinine. Simultaneously, trametinib treatment resulted in a steady increase in the patient’s platelet count (Figure 1B) while leaving his Hgb relatively stable (Figure 1C). The patient tolerated trametinib treatment well, noting an increase in energy within one month of administration. Side effects attributed to therapy included mild alopecia, ankle edema, and intermittent facial erythema and dermatitis.

Overall, the patient's clinical course has been unremarkable, with his WBC counts stabilizing at 10 - 15 x 10^3^/µl and his differential having normalized, indicative of a near-complete hematologic response. At the time of this report, the patient has been on 2 mg of trametinib daily for over 14 months, with his most recent bloodwork from early November 2015 revealing a WBC count of 10 x 10^3^/µl, Hgb of 10.5 g/dL, and a platelet count of 168 x 10^3^/µl.

## Discussion

In this report, we describe a patient with NRAS-G12D-positive aCML who experienced an exceptional response to MEK1/2 inhibition with trametinib. The patient demonstrated rapid improvements in his blood counts and reported symptomatic improvement with an increase in energy level within several months after starting therapy. The response to trametinib has been quite durable with the patient experiencing an ongoing near-complete hematologic response after 14 months of therapy.  

Atypical CML is associated with a poor prognosis, and current therapies have variable clinical success [3, 8]. Hematopoietic stem cell transplantation remains the only potentially curative option but is available for only a minority of patients. To our knowledge, this is the first report of a patient with aCML with an *NRAS* mutation treated with an MEK inhibitor. Although we do not have information regarding the status of the patient’s bone marrow and the current allele burden of the NRAS-G12D mutation, his overall depth and duration of response strongly suggests that targeting the RAS-RAF-MEK-ERK may provide significant hematologic and clinical benefit to patients with aCML and perhaps other hematologic malignancies with *NRAS* mutations. Indeed, an ongoing Phase I/II clinical trial of trametinib has noted promising clinical activity in patients with *RAS*-mutated relapsed/refractory myeloid malignancies [9], and two Phase II clinical trials are underway to evaluate the safety and efficacy of trametinib treatment in combination with an AKT inhibitor for AML and multiple myeloma. When completed, the results of these studies may provide greater insight into the benefits of targeting MEK1/2 in hematologic malignancies.

## Conclusions

This case highlights the potential clinical utility of MEK1/2 inhibition in the treatment of aCML cases harboring *NRAS *mutations. Given the absence of an established standard of care for aCML, this report calls attention to the need for a clinical trial evaluating the safety and efficacy of trametinib in patients with *NRAS*-mutated aCML.

## References

[1] Gotlib J, Maxson JE, George TI, Tyner JW (2013). The new genetics of chronic neutrophilic leukemia and atypical CML: implications for diagnosis and treatment. Blood.

[2] Maxson JE, Gotlib J, Pollyea DA, Fleischman AG, Agarwal A, Eide CA, Bottomly D, Wilmot B, McWeeney SK, Tognon CE, Pond JB, Collins RH, Goueli B, Oh ST, Deininger MW, Chang BH, Loriaux MM, Druker BJ, Tyner JW (2013). Oncogenic CSF3R mutations in chronic neutrophilic leukemia and atypical CML. N Engl J Med.

[3] Zoi K, Cross NC (2015). Molecular pathogenesis of atypical CML, CMML, and MDS/MPN unclassifiable. Int J Hematol.

[4] Eisfeld AK, Schwind S, Hoag KW, Walker CJ, Liyanarachchi S, Patel R, Huang X, Markowitz J, Duan W, Otterson GA, Carson WE 3rd, Marcucci G, Bloomfield CD, de la Chapelle A (2014). NRAS isoforms differentially affect downstream pathways, cell growth, and cell transformation. Proc Natl Acad Sci U S A.

[5] Gilmartin AG, Bleam MR, Groy A, Moss KG, Minthorn EA, Kulkarni SG, Rominger CM, Erskine S, Fisher KE, Yang J, Zappacosta F, Annan R, Sutton D, Laquerre SG (2011). GSK1120212 (JTP-74057) is an inhibitor of MEK activity and activation with favorable pharmacokinetic properties for sustained in vivo pathway inhibition. Clin Cancer Res.

[6] Jing J, Greshock J, Holbrook JD, Gilmartin A, Zhang X, McNeil E, Conway T, Moy C, Laquerre S, Bachman K, Wooster R, Degenhardt Y (2012). Comprehensive predictive biomarker analysis for MEK inhibitor GSK1120212. Mol Cancer Ther.

[7] Burgess MR, Hwang E, Firestone AJ, Huang T, Xu J, Zuber J, Bohin N, Wen T, Kogan SC, Haigis KM, Sampath D, Lowe S, Shannon K, Li Q (2014). Preclinical efficacy of MEK inhibition in Nras-mutant AML. Blood.

[8] Tiu RV, Sekeres MA (2014). Making sense of the myelodysplastic/myeloproliferative neoplasms overlap syndromes. Curr Opin Hematol.

[9] Borthakur G, Popplewell L, Boyiadzis M, Foran JM, Platzbecker U, Vey N, Roland WB, Olin RL, Raza A, Giagounidis A, Ottmann OG, Al-Kali A, Jabbour EJ, Kadia TM, Garcia-Manero G, Bauman JW, Wu Y, Liu Y, Schramek D, Zhu JZ, Wissel P, Kantarjian HM (2015). Phase I/II Trial of the MEK1/2 Inhibitor Trametinib (GSK1120212) in Relapsed/Refractory Myeloid Malignancies: Evidence of Activity in Patients with RAS Mutation-Positive Disease. Blood.

